# Colopexy as a treatment option for the management of acute transverse colon volvulus: a case report

**DOI:** 10.1186/1752-1947-6-151

**Published:** 2012-06-13

**Authors:** Mark J Sage, Jenan Younis, Katie E Schwab, Keith A Galbraith

**Affiliations:** 1 Department of Surgery, Ashford and St Peter’s Hospitals NHS Trust, Guildford Road, Chertsey, Surrey, KT16 0PZ, UK

## Abstract

**Introduction:**

Transverse colon volvulus is an uncommon acute surgical presentation associated with a higher rate of mortality than volvulae at other locations along the colon. Surgical resection or correction is the only treatment, and various methods have been described in case report literature to relieve the volvulus and prevent recurrence.

**Case presentation:**

We present the case of a 25-year-old Caucasian woman who was admitted with a three-day history of abdominal pain, absolute constipation and abdominal distension. Subsequent radiographic and computed tomography imaging revealed right-sided colonic dilatation suggestive of a volvulus. An emergency laparotomy was performed during which the dilated proximal bowel was decompressed and colopexy executed by using the greater omentum to fix the transverse colon at the hepatic and splenic flexures.

**Conclusions:**

Volvulus of the transverse colon is rare but must form part of the clinician's differential diagnosis when encountering a patient with suspected bowel obstruction, especially in younger patients with no previous surgical history. Laparotomy is the treatment of choice and the technique of using the greater omentum as a fixing point for redundant bowel to the lateral abdominal wall is an option that may be considered especially when the bowel appears viable.

## Introduction

Transverse colon volvulus, first reported by Kallio [1] in 1932, is a rare cause of large bowel obstruction but is associated with a greater mortality than the more common sigmoid or caecal volvulae. Several surgical options to prevent recurrence have been described in the literature but we believe this is the first case where the greater omentum has been used as a fixation point for the transverse colon.

### Case presentation

A 25-year-old Caucasian woman presented to our emergency department with a three-day history of abdominal pain, distension and absolute constipation with vomiting. There was no significant medical or surgical history. She took no regular medications, and was a non-smoker and non-drinker. Bedside observations were normal. A physical examination revealed a distended tympanic abdomen with tenderness across the lower abdomen but no signs of peritonism. A urine dipstick test was unremarkable and blood tests showed a raised white blood cell count (14.4 ×109 cells/L) and C-reactive protein (35mg/L). Urea and electrolytes, hemoglobin and liver function tests were all normal.

**Figure 1:**
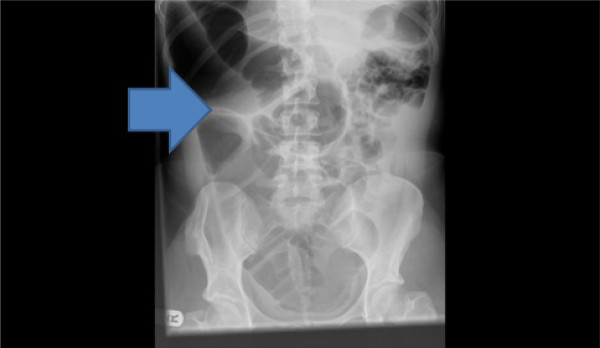
Radiograph of the abdomen showing gross dilatation of the right side of the colon.

**Figure 2:**
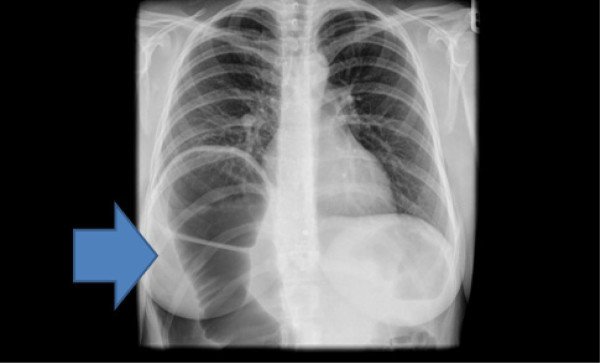
Radiograph of the chest showing gross dilatation of the right side of the colon with Chilaiditi’s sign.

**Figure 3:**
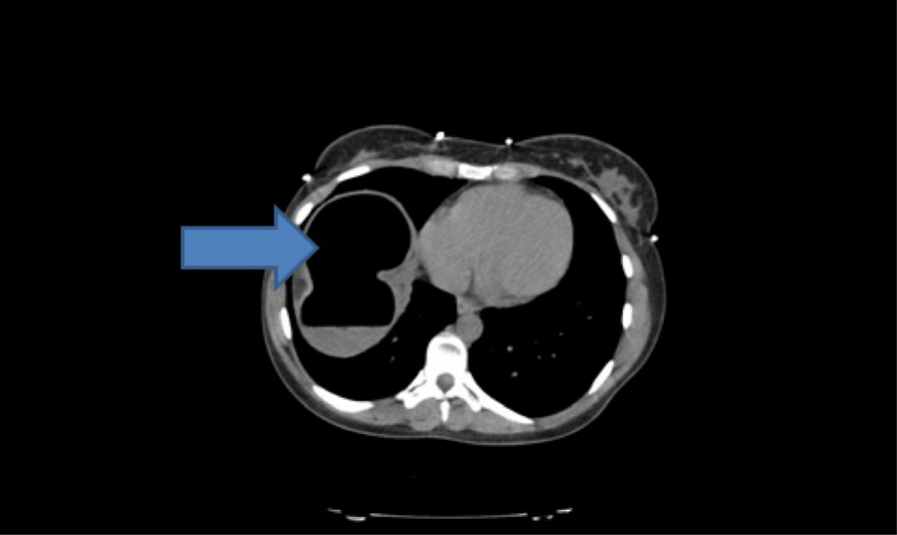
Computed tomography scan of the abdomen showing massive dilatation of the caecum, which measured greater than 14 cm in diameter.

**Figure 4:**
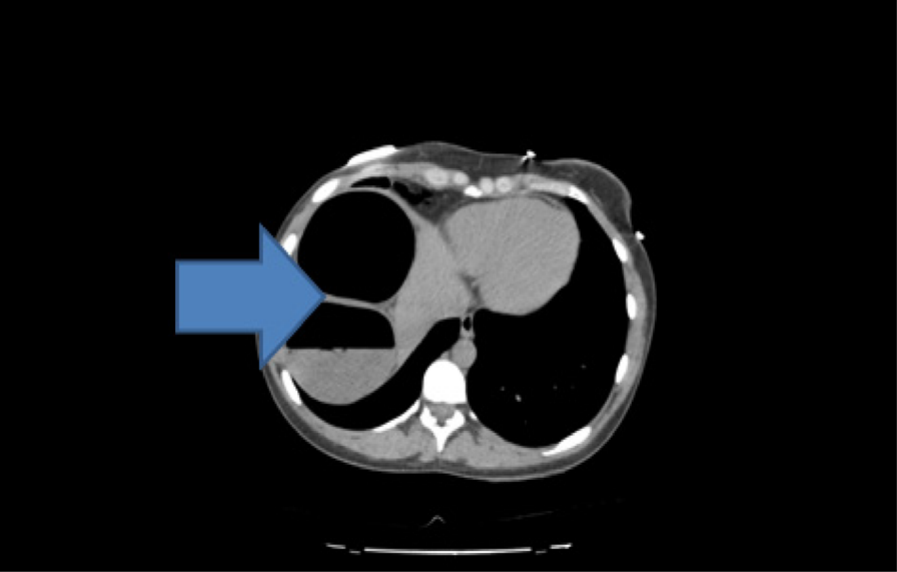
Computed tomography scan of the abdomen showing massive dilatation of the caecum, which measured greater than 14 cm in diameter.

**Figure 5:**
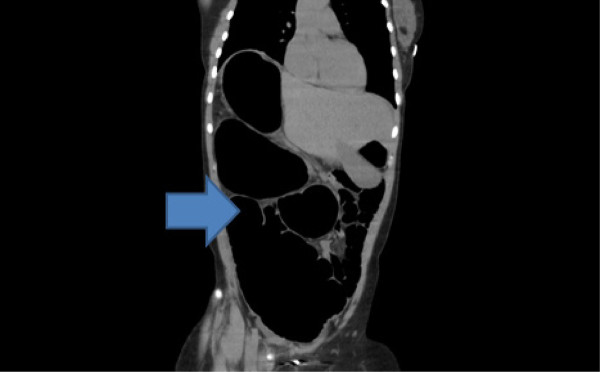
Computed tomography scan of the abdomen showing the distal colon collapsed with a transition point in the mid abdomen suggestive of a volvulus or band.

Radiographs of the abdomen (Figure 1) and chest (Figure 2) showed gross dilatation of the right side of the colon with Chilaiditi’s sign. A computed tomography (CT) scan of the abdomen showed massive dilatation of the caecum, which measured greater than 14 cm in diameter (Figures 3, 4, and 5). The distal colon was collapsed with a transition point in the mid abdomen suggestive of a volvulus or band.

A nasogastric tube was inserted, intravenous fluid resuscitation commenced and our patient underwent an emergency exploratory laparotomy. Intra-operatively, the bowel was found to be significantly dilated from the caecum to the distal transverse colon and the distal small bowel was mildly dilated, giving the impression of decompression through an incompetent ileocaecal valve. There was a volvulus of the transverse colon in the right upper quadrant. The transverse mesocolon appeared to take origin from the right upper quadrant as opposed to the usual location as an extension of the peritoneum from the posterior abdominal wall centrally. There was no evidence of colonic ischemia or serosal injury.

The large bowel was decompressed with a 14 gauge needle allowing the tightly held bowel to be manipulated more easily. The large bowel was fully unraveled and the volvulus corrected then decompressed distally into the descending colon. The greater omentum on the transverse colon was then fixed at both the hepatic and splenic flexures by creating pouches in the lateral anterior abdominal walls, and fixing the omentum within this with polydioxanone sutures (PDS). The central portion of the omentum was purposefully caught in the mass closure of the wound to ensure additional central fixation.

Post-operatively our patient made a good recovery. By day five she was tolerating a full diet and was discharged six days post-operatively. Despite a superficial wound infection at two weeks, she made a full recovery with no complications noted at her three month and six month follow-up.

## Discussion

Volvulus of the transverse colon is a rare surgical emergency and as of 2008 there were only 100 published cases worldwide. Transverse volvulus accounts for only 2% to 4% of cases of colonic volvulus, but carries a 33% mortality rate [2,3]. It occurs most often in the second and third decades of life with a second peak in the seventh decade and is more common in women [2]. Up to 50% of patients report experiencing similar symptoms in the past [4]. A volvulus is caused by the twisting of the colon on its vascular pedicle causing venous obstruction, followed by arterial compromise and, potentially, ischemia [5,6].

Etiology can be acquired or congenital in nature [5-7].

The former commonly is due to adhesions, inflammatory strictures, carcinoma or malposition of the colon following previous surgery [5-8].

Congenital abnormalities include midgut malrotation, resulting in abnormal fixation [2,4,8], congenital megacolon [8], elongation and redundancy of the transverse colon and narrowing, absence or malfixation of the mesenteries and their attachments [9], this being the etiology observed in our patient’s case.

Transverse colon volvulus has been described in the literature as subacute-progressive or fulminating [10]. Fulminating volvulus is a more aggressive form, rapidly progressing due to closed loop obstruction resulting in vascular compromise [4]. The subacute form, presents with more subtle signs of obstruction [5,6].

Radiologically, it is classic to find a ‘bent inner tube’ sign on plain abdominal films [5] or a ‘bird’s beak’ deformity on contrast enema [7].

With regards to management, in contrast to sigmoid volvulus, which can often be decompressed during sigmoidoscopy, transverse colon volvulus must be surgically corrected [11]. When necrosis has occurred, resection of the non-viable tissue may take the form of resection with primary anastamosis or resection with colostomy or ileostomy and mucous fistula [5]. However, many authors advocate segmental transverse colectomy or an extended right colectomy as the treatment of choice, even in the event of the bowel being viable, as it carries virtually no risk of recurrence when compared to colopexy which has a reported risk of 30% to 75% of recurrence. Indeed it has also been documented that it is not uncommon that such patients have presented previously with self-limiting episodes of subacute obstruction. This is thought to be due to the intermittent volvulus of the transverse colon [4].

The argument in favour of colopexy over resection for viable bowel resides in eliminating the risks associated with the latter option. These include risks of anastomotic leak, paralytic ileus, stenosis and the need for a stoma; all of which carry considerable morbidity as well as mortality [12]. Thus it would appear that colopexy appears the safer short-term option for the patient with viable bowel intra-operatively, whilst resection could potentially be preferable in the longterm. The wide recurrence rate reported is dated, however, and no colopexy technique used is entirely identical. This makes it difficult at present to fully justify a resection in such a group of patients [13]. The clear limitation with our patient’s case is that whilst we have seen our patient’s recovery six months from surgery, the long-term success of the technique described here is unknown.

## Conclusions

While rare, transverse colon volvulus must form part of the clinician’s differential diagnosis when encountering a patient with suspected bowel obstruction, especially in younger patients with no previous surgical history. Exploratory laparotomy allows full identification of the pathology and access for corrective and restorative management. The technique of fixing the greater omentum in anterior abdominal wall pouches to correct anatomy has been demonstrated here as a successful method of treatment in the shortterm that may be considered.

## Consent

Written informed consent was obtained from the patient for publication of this case report and accompanying images. A copy of the written consent is available for review by the Editor-in-Chief of this journal.

## Competing interests

The authors declare that they have no competing interests.

## Authors’ contributions

MS was involved in the initial diagnosis and management of our patient upon presentation to hospital, and in reviewing the literature and writing the initial manuscript. JY and KS were in theatre at the laparotomy and were major contributors to the manuscript and edited its contents. KG was the senior clinician involved in overseeing the care of our patient and approved the final version of the manuscript. All authors read and approved the final manuscript.
